# Ndt80 Orchestrates Copper Stress Responses and Mitochondrial Homeostasis in *Candida albicans*

**DOI:** 10.3390/jof12040294

**Published:** 2026-04-20

**Authors:** Hsuan-Yu Chen, Hsiu-Jung Lo, Chi-Jan Lin, Chung-Yu Lan

**Affiliations:** 1 Institute of Molecular and Cellular Biology, National Tsing Hua University, Hsinchu 300044, Taiwan; wh40624@gmail.com; 2 Taiwan Mycology Reference Center, National Institute of Infectious Diseases and Vaccinology, National Health Research Institutes, Miaoli County 350401, Taiwan; hjlo@nhri.org.tw; 3 School of Dentistry, China Medical University, Taichung 404328, Taiwan; 4 Institute of Molecular Biology, National Chung Hsing University, Taichung 402202, Taiwan; 5 Department of Life Science, National Tsing Hua University, Hsinchu 300044, Taiwan; 6 School of Medicine, National Tsing Hua University, Hsinchu 300044, Taiwan

**Keywords:** *C*. *albicans*, Ndt80, copper homeostasis, copper tolerance, mitochondrial functions

## Abstract

Copper is a crucial cofactor that sustains multiple cellular electron-transfer reactions, making it an essential element for life. However, cytotoxic levels of copper can cause structural damage and cell death through the production of reactive oxygen species (ROS) and nonspecific attacks on proteins. Moreover, immune cells, including neutrophils and macrophages, accumulate copper to induce oxidative bursts that kill engulfed pathogens. Therefore, a well-regulated copper homeostasis system is required for the human commensal fungus *Candida albicans* to thrive in extreme host environments. Remarkably, *C. albicans* exhibits higher copper tolerance than the nonpathogenic model yeast *Saccharomyces cerevisiae*, suggesting the presence of a specific copper tolerance mechanism that supports its adaptability to copper stress. Ndt80 is a versatile transcription factor that regulates several biological processes in *C. albicans*, ranging from morphological control to drug resistance. This study further reveals that Ndt80 may contribute to copper tolerance by regulating copper transporters and copper-dependent superoxide dismutases (Sods). Additionally, RNA sequencing and complementary approaches uncovered the involvement of Ndt80 in plasma membrane integrity and mitochondrial respiration under copper stress, further linking Ndt80 to copper tolerance. Together, these results broaden our understanding of Ndt80 functions and provide new insights into copper tolerance in *C. albicans*.

## 1. Introduction

Copper is an essential trace metal required by almost all living organisms to support diverse biochemical processes [1,2]. Its ability to switch between the cupric (Cu^+2^) and cuprous (Cu^+^) states makes copper a crucial cofactor for numerous cellular enzymes, including superoxide dismutases (Sods) involved in antioxidant defense and components of the mitochondrial electron transport chain (ETC) required for energy production [3,4]. Copper is also closely linked to iron homeostasis. In fungal pathogens, copper-dependent enzymes, such as the multicopper ferroxidase Fet3 in *Candida albicans* and the multicopper oxidoreductase FetC in *Aspergillus fumigatus*, catalyze the oxidation of ferrous (Fe^+2^) ions, thereby facilitating iron uptake from the host environment [5,6,7]. Similarly, in mammalian cells, copper regulates iron loading onto transferrin, the major iron transport protein in the bloodstream [8]. In *Cryptococcus neoformans*, copper is additionally required for biosynthesis of melanin, a key virulence factor that enhances fungal resistance to environmental stress and phagocytosis of host immune defenses [9]. Together, these findings highlight the central role of copper in host–pathogen interactions and underscore the importance of copper homeostasis for fungal survival and pathogenicity [10].

Despite its essential roles, excess copper is highly cytotoxic in *C*. *albicans*. Copper catalyzes Fenton-like reactions that generate reactive oxygen species (ROS), leading to damage of proteins, lipids, and DNA. It can also disrupt iron-sulfur (Fe-S) clusters, impairing protein structure and function. Recent studies further link copper toxicity to cuproptosis, a distinct form of cell death driven by mitochondrial copper accumulation and loss of Fe-S cluster proteins [11]. At the host–pathogen interface, immune cells exploit copper cytotoxicity as an antifungal defense mechanism. During infection, macrophages and neutrophils transport copper into phagolysosomes via the copper-transporting ATPase ATP7A, promoting oxidative stress and pathogen killing [12,13,14,15]. These observations illustrate the dual nature of copper as both an essential micronutrient and a potent cytotoxin, emphasizing the need for tight copper homeostasis [1,11,16,17].

*C. albicans* possesses sophisticated stress–response systems and virulence traits that enable adaptation to host environments. Compared with the nonpathogenic yeast *Saccharomyces cerevisiae*, *C. albicans* exhibits significantly higher tolerance to elevated copper levels and even to copper surfaces [18,19]. This enhanced tolerance suggests the existence of specialized regulatory mechanisms that protect against copper-mediated oxidative stress imposed by host immune defenses. Understanding these mechanisms may, therefore, provide insights into fungal virulence and reveal potential antifungal targets.

The Ndt80 family consists of conserved fungal transcription factors with diverse biological functions. In *S. cerevisiae*, Ndt80 activates the genes required for meiosis and sporulation by binding the middle sporulation element (MSE) in target promoters [20,21,22]. In *C. albicans*, three Ndt80-like proteins, Ndt80, Rep1, and Ron1, have been identified. These paralogs regulate hyphal development, biofilm formation, and antifungal drug resistance, with Ndt80 and Ron1 coordinately controlling *N*-acetylglucosamine (GlcNAc)-induced hyphal growth [23,24]. Genome-wide ChIP-chip analyses indicate that Ndt80 associates with approximately 23% of *C. albicans* gene promoters, suggesting a broad role of Ndt80 in transcriptional regulation [25,26]. Consistent with this notion, phenotypic profiling demonstrated that the *ndt80* deletion (*ndt80*Δ/Δ) mutant displays altered responses to multiple environmental stresses, including increased sensitivity to copper [27]. However, the molecular mechanisms underlying Ndt80-mediated copper stress responses remain unclear.

In this study, we investigated the role of Ndt80 in copper homeostasis and its impact on cellular functions affected by copper toxicity. Growth assays under varying CuSO_4_ concentrations were used to evaluate copper sensitivity, while membrane integrity and Cu-dependent Sod activation were analyzed to assess stress responses. We further examined mitochondrial function in the *ndt80*Δ/Δ mutant. Our findings demonstrate that Ndt80 plays an important role in copper homeostasis in *C. albicans* and contributes to maintaining mitochondrial functional integrity under copper stress.

## 2. Materials and Methods

### 2.1. C. albicans Strains and Growth Conditions

The *C. albicans* strains used in this study are listed in Table 1. Cells were routinely stored at −80 °C. Before each experiment, cells were streaked onto YPD agar plates (1% yeast extract, 2% peptone, 2% glucose, 1.5% agar) and incubated at 30 °C for 24 h.

To assess copper-dependent growth, a single colony was inoculated into YPD broth and incubated overnight (~16 h) at 30 °C with shaking at 180 rpm. Overnight cultures were diluted to 1.5 × 10^5^ cells/mL in 200 μL of YPD medium supplemented with 0.8 mM bathocuproinedisulfonic acid disodium salt (BCS; copper-restricted condition), no supplement (low-copper condition), or various concentrations of CuSO_4_ (10, 12, and 15 mM; high-copper conditions). Cells were loaded into 96-well flat microplates and incubated at 30 °C for 3 d. Growth was monitored hourly by measuring the optical density at 600 nm (OD_600_) using a SPECTROstar Nano plate reader (BMG Labtech, Ortenberg, Germany).

For other assays, cells from overnight cultures were subcultured in YPD medium with or without 10 mM CuSO_4_ (starting at ~1.5 × 10^6^ cells/mL) and grown at 30 °C with shaking until reaching the exponential phase. Cells were harvested by centrifugation and washed twice with phosphate-buffered saline (PBS) before further experiments. Unless otherwise stated, reagents were purchased from Sigma-Aldrich (St. Louis, MO, USA).

### 2.2. Inductively Coupled Plasma–Mass Spectrometry (ICP-MS)

To measure intracellular copper levels, cells were subcultured in YPD medium with or without 10 mM CuSO_4_ for 5 h, harvested by centrifugation, and washed three times with sterile double-distilled water (ddH_2_O). Cell pellets were resuspended in 200 μL of 70% nitric acid and incubated at room temperature for 24 h, followed by dilution with 800 μL ddH_2_O. Samples were further diluted with 1% (*v*/*v*) nitric acid in 15 mL conical tubes (pre-rinsed with 20% nitric acid). Internal calibration standards (4, 10, 20, and 40 ppm) were prepared from a 1000 ppm high-purity copper solution with 1% nitric acid. Copper concentrations were quantified using an iCAP TQ ICP-MS system (Thermo Scientific, Waltham, MA, USA) and normalized based on internal standards.

### 2.3. RNA Extraction, Reverse Transcription (RT), and Real-Time Quantitative PCR (qPCR)

Cells were harvested after 5 h of subculture in YPD, with or without 10 mM CuSO_4_ at 30 °C, and stored at −80 °C until use. Total RNA extraction and RT were performed as previously described [29]. Real-time qPCR reactions were conducted using 30 ng cDNA on a StepOne Plus real-time PCR system (Applied Biosystems, Waltham, MA, USA). The primers used in this study are listed in Table S1 in the Supplementary Materials. The *ACT1* transcript was used as an internal control for normalization. Relative expression levels were calculated using the 2^−∆∆CT^ method [30].

### 2.4. RNA Sequencing (RNA-Seq) and Data Analysis

Cells were subcultured in YPD broth, with or without 10 mM CuSO_4_, for 5 h at 30 °C. Total RNA from three biological replicates was extracted using a NautiaZ Bacteria/Fungi RNA Mini kit (Nautia Gene, Taipei, Taiwan). Libraries were prepared using the TruSeq Stranded mRNA Library Prep Kit (Illumina, San Diego, CA, USA). Briefly, mRNA was isolated from 1 µg of total RNA using oligo(dT)-coupled magnetic beads, fragmented at elevated temperature, and reverse transcribed into the first-strand cDNA using random primers. After the synthesis of double-strand cDNA, 3′-end adenylation and adapter ligation were performed. The resulting cDNA fragments were enriched by PCR and purified using the AMPure XP system (Beckman Coulter, Beverly, MA, USA). Library quality was assessed using a Qsep400 Bio-Fragment Analyzer (Bioptic Inc., New Taipei City, Taiwan) and quantified with a Qubit 2.0 Fluorometer (Thermo Scientific, Waltham, MA, USA). Sequencing was conducted on an Illumina Novaseq X Plus platform (Illumina, San Diego, CA, USA) by Genomics, BioSci & Tech Co. (New Taipei City, Taiwan), generating 150 bp paired-end reads.

The processed sequence reads were aligned to the *C. albicans* SC5314_A22 genome using the Bowtie 2 alignment tool (version 2.3.2) and the read count control tool RSEM (version 1.2.31) [31,32,33]. Raw read counts were analyzed to identify differentially expressed genes (DEGs) using the PyDESeq2 package (version 0.4.12) implemented in Python (version 6.0.5). Default parameters were applied based on the workflow described in the PyDESeq2 documentation (https://pydeseq2.readthedocs.io/en/stable/auto_examples/plot_step_by_step.html#sphx-glr-auto-examples-plot-step-by-step-py, accessed on 21 November 2024), with some modifications. RNA-seq data are available in the NCBI Sequence Read Archive (SRA) under accession number PRJNA1414925. Transcriptome analyses were performed using three independent biological replicates of *C. albicans* WT and *ndt80*Δ/Δ strains cultured in YPD medium, with or without 10 mM CuSO_4_.

To adjust for differences in sequencing depth among the samples, read counts were normalized to size factors derived from the median ratio of gene counts relative to a pseudo-reference (i.e., the geometric mean of gene counts across all samples). To model variability in the read counts across biological replicates, normalized read counts were fitted to a trend curve derived from dispersion estimation using a negative binomial distribution. Log_2_ fold change (LFC) values were calculated by comparing the *ndt80*Δ/Δ mutant to the WT strain under conditions, with or without 10 mM CuSO_4_, and LFC shrinkage was applied to reduce variance associated with low-count genes. The Wald test was used to compute the *p*-values (*P*) for statistical significance of each gene. To control the false discovery rate (FDR) arising from multiple testing, adjusted *p*-values (adjusted *P*) were calculated using the Benjamini–Hochberg procedure. DEGs were defined as those with an adjusted *p*-value < 0.05 and a fold change > 1.5 (log_2_ fold change > 0.585). All upregulated and downregulated DEGs are listed in Table S2. A hierarchical heatmap was generated based on the gene expression patterns of DEGs across biological triplicates. Expression levels were transformed into a *z*-score, calculated as $z=\frac{x-\mu }{\sigma }$, where μ represents the mean read count across all samples, and σ represents the standard deviation. The *z*-scores and gene clustering were generated using the ‘clustermap’ function in the Seaborn library (version 0.13.2), implemented in Python and visualized as a heatmap (Figure S1).

DEGs were subjected to Gene Ontology (GO) enrichment analysis using the Protein ANalysis THrough Evolutionary Relationships (PANTHER) Overrepresentation Test, with significance assessed by a binomial test and FDR correction at a cutoff of 0.05 [34,35]. Complete GO enrichment results for both upregulated and downregulated DEGs are provided in Table S3. For Gene Set Enrichment Analysis (GSEA), normalized read counts in GCT format, generated using the R DESeq2 package (version 4.5.0), were used as input for the GSEA software (version 4.4.0). Gene set collections were obtained from the *Candida* Genome Database (CGD), provided by Andre Nantel. All significantly enriched gene sets identified by GSEA are summarized in Table S4. Interaction networks of GSEA-enriched gene sets were visualized using Cytoscape (version 3.10.3) following the EnrichmentMap pipeline available on the website (https://cytoscape.org/cytoscape-tutorials/protocols/enrichmentmap-pipeline/#/, accessed on 18 May 2025). Networks are created with a *p*-value cutoff of 0.01, an FDR *q*-value cutoff of 0.05, and a similarity cutoff of 0.5 (overlap coefficient), as described previously [36].

### 2.5. Regulatory Sequence Analysis of DEGs

To determine whether the Ndt80-binding motif, also known as the middle sporulation element (MSE), was present in the promoter regions of DEGs identified under high-copper conditions, promoter sequences of these DEGs were retrieved and blasted with consensus sequences from PathoYeastract *C. albicans* (https://yeastract-plus.org/pathoyeastract/calbicans/index.php, accessed on 6 February 2026) [37]. MSE consensus sequences were obtained from previous studies [28,38,39,40]. DEGs containing the predicted MSE motifs within their promoter regions are listed in Table S5.

### 2.6. Cell Susceptibility to Plasma Membrane-Disrupting Agents

Cell pellets were harvested after growth in a YPD medium at 30 °C and washed twice with PBS. Cells were adjusted to a density of 3 × 10^7^ cells/mL in PBS and subjected to 10-fold serial dilutions. To assess cell susceptibility to plasma membrane-disrupting agents under copper stress, YPD agar plates supplemented with either 5 Mm or 10 mM CuSO_4_ were prepared containing 0.09% sodium dodecyl sulfate (SDS). Five microliters of each dilution were spotted onto the agar plates, which were incubated at 30 °C for 3 d and photographed daily.

### 2.7. Measurement of Plasma Membrane Potential (ΔΨ) and Permeability

Cells were harvested after subculture in a YPD medium with or without 10 mM CuSO_4_ for 5 h at 30 °C. For measurement of plasma membrane ΔΨ and permeability, cells (1.125 × 10^8^ cells/mL) were resuspended in 200 μL of PBS containing either 20 μg/mL DiBAC_4_(3) (Invitrogen, Carlsbad, CA, USA) or 25 nM SYTOX Green (Invitrogen) and incubated at 30 °C for 30 min [41]. Following staining, cells were collected and washed two to three times with PBS. Fluorescence intensity was measured using a CytoFLEX flow cytometer (Beckman Coulter, Brea, CA, USA) equipped with a FITC detector. Data were analyzed using CytExpert software (version 2.5).

### 2.8. Assessment of Lipid Peroxidation and Lipid Droplet Accumulation

To assess lipid peroxidation, the thiobarbituric acid reactive substances (TBARS) assay was performed as previously described [42]. Briefly, cells were harvested after 5 h of subculture in a YPD medium, with or without 10 mM CuSO_4_ at 30 °C, washed, and resuspended in 200 μL of PBS. Cells were then lysed by vortexing with glass beads for 30 s, followed by cooling on ice for 30 s; this process was repeated 10 times. Cell debris was removed by centrifugation at 4 °C for 10 min, and the supernatant was collected for analysis using the TBARS (TCA method) Assay Kit (Cayman Chemical, Ann Arbor, MI, USA) according to the manufacturer’s instructions. Malondialdehyde (MDA), a major product of lipid peroxidation, forms a complex with thiobarbituric acid (TBA) under high-temperature (90–100 °C) and acidic conditions. The resulting MDA-TBA adduct was measured colorimetrically at 535 nm using a SPECTROstar Nano plate reader (BMG Labtech, Ortenberg, Germany).

Lipid droplet accumulation was measured using Nile red staining as previously described [41]. After 5 h of subculture in a YPD medium, with or without 10 mM CuSO_4_, at 30 °C, cells (1.125 × 10^8^ cells/mL) were resuspended in 200 μL of PBS containing 5 μg/mL of Nile red and incubated at 30 °C for 30 min. Cells were subsequently collected and washed two to three times with PBS. Fluorescence intensity was measured using a CytoFLEX flow cytometer (Beckman Coulter, Brea, CA, USA) equipped with a PC5.5 detector. Data analysis was conducted using CytExpert software.

### 2.9. Measurement of Cellular and Mitochondrial ROS

Cells were harvested after 5 h of subculture in a YPD medium, with or without 10 mM CuSO_4_, at 30 °C, and washed twice with PBS. For measurement of total cellular ROS, cells (1.125 × 10^8^ cells/mL) were resuspended in 200 μL of PBS containing either 20 μM dihydroethidium (DHE) or 20 μg/mL of 2′-7′-dichlorodihydrofluorescein diacetate (H_2_DCFDA) and incubated at 30 °C for 10–20 min [41]. For measurement of mitochondrial ROS, cells (1.125 × 10^8^ cells/mL) were resuspended in 200 μL of PBS containing 5 μM MitoSOX Red (Invitrogen, Carlsbad, CA, USA) and incubated at 30 °C for 10–20 min [41]. Following staining, cells were washed two to three times with PBS. Fluorescence intensity was measured using a CytoFLEX flow cytometer (Beckman Coulter, Brea, CA, USA) equipped with PE and FITC detectors. Data were processed using CytExpert software.

### 2.10. Protein Extraction and Detection of Hog1 Phosphorylation by Western Blot

Protein extraction was conducted as previously described [43]. Cells were harvested after 5 h of subculture in a YPD medium, with or without 10 mM CuSO_4_, at 30 °C, and washed twice with PBS. Cell pellets were lysed with acid-washed glass beads in Radio-Immunoprecipitation Assay (RIPA) lysis buffer supplemented with a protease inhibitor cocktail (Abcam Limited, Cambridge, UK) and phosphatase inhibitors. Total protein was extracted by vigorous vortexing with glass beads and collected by centrifugation. Protein concentrations were determined using the Bio-Rad Bradford protein assay (Bio-Rad, Hercules, CA, USA).

Equal amounts of proteins (25 μg) were subjected to 10% SDS-polyacrylamide gel electrophoresis and transferred to PVDF membranes [43]. Phosphorylated Hog1 (Hog1-p) and total Hog1 were detected using an anti-phospho-p38 MAPK (catalog no. 9215, Cell Signaling Technology, Danvers, MA, USA) and an anti-Hog1 primary antibody (catalog no. sc-9079, Santa Cruz Biotechnology, Santa Cruz, CA, USA), respectively. Horseradish peroxidase (HRP)-conjugated anti-rabbit IgG (catalog no. GTX213110-01, GeneTex, Hsinchu, Taiwan) was used as the secondary antibody. Protein bands were visualized using Western Lightning Plus chemiluminescent substrates (PerkinElmer, Waltham, MA, USA), and signals were captured with a ChemiDoc MP Imaging System (Bio-Rad, Hercules, CA, USA). Band intensities were quantified using ImageJ software (version v1.54g). Total Hog1 was used as the loading control, and Hog1 phosphorylation levels were expressed as the ratio of Hog1-P to total Hog1.

### 2.11. Cell Rescue Assay by Inhibition of Mitochondrial Respiration

Cells were cultured in a YPD medium, harvested, and washed twice with PBS. Cells (3 × 10^7^ cells/mL) were resuspended in 1 mL of PBS and subjected to 10-fold serial dilutions. To determine whether inhibition of mitochondrial ETC activity alleviates copper toxicity, YPD agar plates containing 10 mM CuSO_4_ were supplemented with either 10 µM rotenone (complex I inhibitor) or 5 μM antimycin A (complex III inhibitor). Five microliters of each dilution were spotted onto the plates, which were then incubated at 30 °C for 3 d and photographed daily.

### 2.12. Measurement of Mitochondrial Membrane Potential (ΔΨ)

Mitochondrial ΔΨ was measured as previously described [41]. After subculture in a YPD medium, with or without 10 mM CuSO_4_, at 30 °C, cells were harvested and washed twice with PBS. Cells (1.125 × 10^8^ cells/mL) were resuspended in 200 μL of PBS containing either 25 μM Rhodamine 123 or 2 μM JC-1 and incubated at 30 °C for 10–15 min. After staining, cells were collected and washed two to three times with PBS. Fluorescence intensity was measured using a CytoFLEX flow cytometer (Beckman Coulter, Brea, CA, USA) equipped with PE and FITC detectors. Data were evaluated using CytExpert software.

### 2.13. Measurement of Intracellular ATP

Intracellular ATP levels were measured as previously described [41]. Cells (6 × 10^8^ cells/mL) were harvested after 5 h of subculture in a YPD medium, with or without 10 mM CuSO_4_, at 30 °C, washed twice with PBS, and lysed using glass beads. ATP levels were quantified using the ATP Determination Kit (A22066, Invitrogen, Carlsbad, CA, USA) according to the manufacturer’s instructions. A reaction buffer containing 0.5 mM D-luciferin, 1.25 μg/mL of firefly luciferase, and 1 mM dithiothreitol (DTT) was freshly prepared for each experiment. Cell lysates (10 µL) were mixed with 990 µL of reaction buffer, and 200 µL of the mixture was transferred to a white 96-well microplate. Luminescence at 560 nm was measured using a VICTOR Nivo multimode microplate reader (PerkinElmer, Shelton, CT, USA). ATP concentrations were calculated based on a standard curve generated using ATP concentrations of 0, 10, 100, and 1000 nM.

### 2.14. Measurement of Oxygen Consumption Rate (OCR)

OCR was measured as previously described with slight modifications [44]. Cell pellets were harvested and washed after subculture as described above. For the OCR measurement, cells (3 × 10^6^ cells/mL) were resuspended in PBS. Prior to the assay, the high-resolution respirometry Oroboros Oxygraph-O2k (Oroboros Instruments, Innsbruck, Austria) was air-calibrated at 30 °C using PBS by following the manufacturer’s instructions. Each chamber was then loaded with 2.1 mL of cell suspension, and the OCR was recorded until a stable baseline signal was achieved. To evaluate the respiration parameters, 2 μL of 150 mM triethyltin bromide (TET) and 2 μL of 2 mM antimycin A were sequentially injected into each chamber to assess the baseline cellular OCR and non-mitochondrial respiration, respectively. Basal respiration was calculated by subtracting non-mitochondrial respiration from the baseline cellular OCR. Proton leak respiration was determined as the difference between baseline cellular OCR and OCR following the TET addition. ATP-linked respiration was calculated by subtracting proton leak respiration from basal respiration.

### 2.15. Statistical Analysis

Significant difference between samples was determined using two-tailed Student’s *t*-tests. Differences were considered significant at *p* < 0.05.

## 3. Results

### 3.1. The ndt80Δ/Δ Mutant Is Hypersensitive to High-Copper Conditions

**Figure 1:**
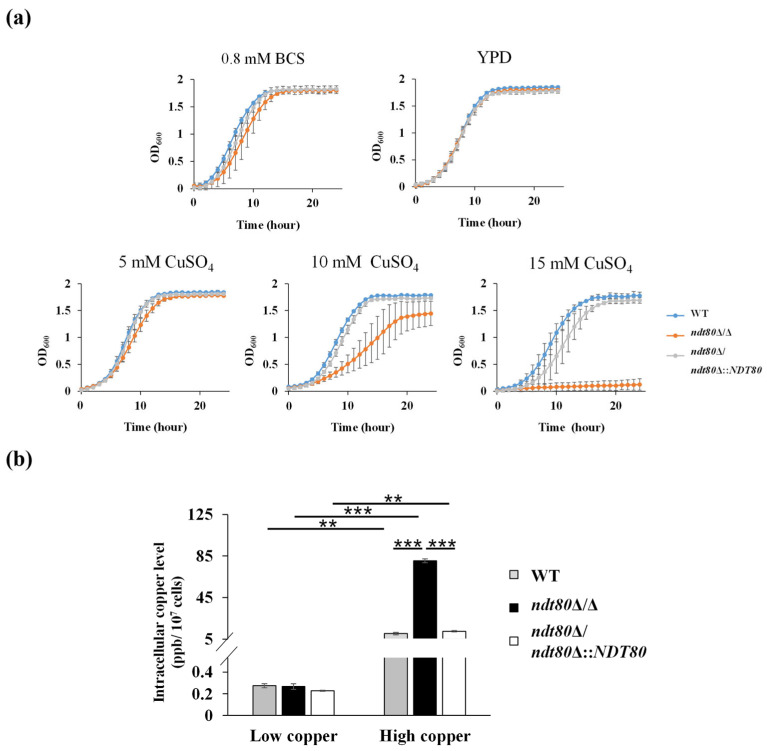
The *ndt80*Δ/Δ mutant is hypersensitive to high-copper conditions. (**a**) Cell growth under copper-restricted, low-copper, and high-copper conditions at 30 °C. Data were collected from three independent experiments and are presented as the mean ± standard deviation (SD). (**b**) Intracellular copper levels measured by ICP-MS. Results are shown as the mean ± SD from three independent experiments. ** *p* < 0.01; *** *p* < 0.001.

To examine the relationship between Ndt80 and copper homeostasis, cells were grown in copper-restricted (YPD supplemented with BCS), low-copper (YPD alone), and high-copper (YPD supplemented with CuSO_4_) conditions. As shown in Figure 1a, no significant differences in cell growth were observed among all tested strains under copper-restricted or low-copper conditions. In contrast, the *ndt80*Δ/Δ mutant exhibited markedly increased sensitivity to elevated copper concentrations compared with the WT and *NDT80*-reintegrated strains. To further investigate the role of Ndt80 in cellular copper regulation, intracellular copper levels were quantified by ICP-MS. Under low-copper conditions, all strains contained similarly low levels of intracellular copper. However, under high-copper conditions (10 mM CuSO_4_), the *ndt80*Δ/Δ mutant accumulated significantly more intracellular copper levels than the control strains (Figure 1b).

**Figure 2:**
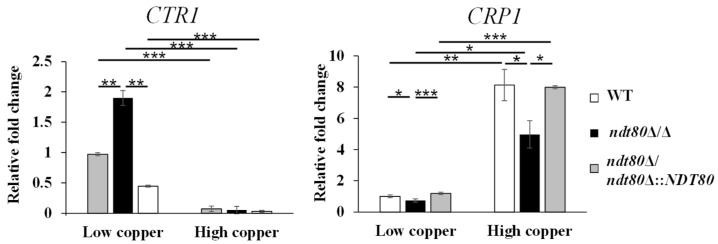
Expression of copper transporter genes under low- and high- copper conditions. Gene expression levels were determined by real-time qPCR. Results were collected from three independent experiments and are presented as the mean ± SD. * *p* < 0.05; ** *p* < 0.01; *** *p* < 0.001.

Ctr1 and Crp1 are copper transporters involved in copper homeostasis. Ctr1 mediates copper uptake, whereas Crp1 functions in copper export under low- and high-copper conditions, respectively [13,19,45,46,47]. Given the elevated intracellular copper levels observed in the *ndt80*Δ/Δ mutant (Figure 1b), expression of *CTR1* and *CRP1* was examined by real-time qPCR analysis. As expected, *CTR1* expression predominated under low-copper conditions, whereas *CRP1* was strongly induced under high-copper conditions (Figure 2). Notably, the *ndt80*Δ/Δ mutant exhibited a different expression pattern of both copper transporters compared with the WT and *NDT80*-reintegrated strains under low- and high-copper conditions, respectively. Collectively, these results indicate that Ndt80 plays an important role in regulating cellular copper homeostasis and tolerance.

### 3.2. Transcriptome Profiling of the ndt80Δ/Δ Mutant in Response to Copper Conditions

**Figure 3:**
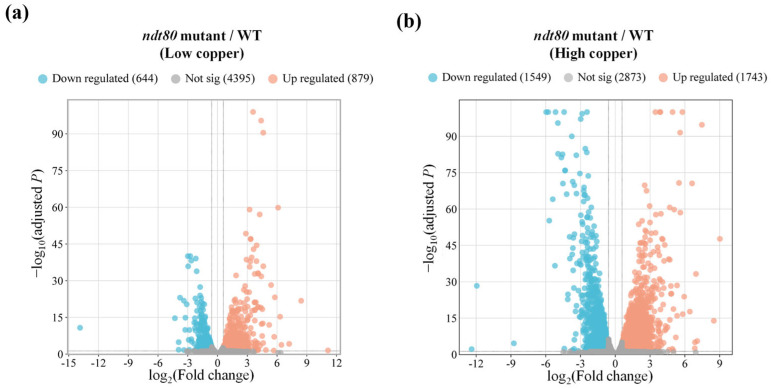
Transcript profiling of the *ndt80*∆/∆ mutant under low- and high-copper conditions. Differentially expressed genes (DEGs) in the *ndt80*Δ/Δ mutant compared with the WT strain under (**a**) low-copper conditions and (**b**) high-copper conditions. DEGs were defined by an adjusted *p* < 0.05 and an expression fold change greater than 1.5, as shown in the volcano plots. Two vertical lines are used to set boundaries for upregulated (**right**) and downregulated (**left**) genes.

To obtain a global view of the impacts of *NDT80* deletion on copper responses, RNA-seq analysis was performed to identify DEGs in the *ndt80*Δ/Δ compared to the WT strain under low- and high-copper conditions. DEGs were defined as the expression change of at least 1.5-fold with an adjusted *p*-value < 0.05. Volcano plots revealed numerous significantly upregulated and downregulated genes in the *ndt80*Δ/Δ mutant under both conditions (Figure 3a,b). All identified DEGs are listed in Table S2. Additionally, to highlight the modulation by Ndt80 under high-copper conditions, we performed copper-responsive transcriptomic profiling in the wild-type strain, presented as a hierarchical clustering heatmap (Figure S1). Under high-copper conditions, several genes associated with copper and plasma membrane function were upregulated, including *FET31*, *FET33*, *FET99*, *SEF2,* and *DRS2*. These genes encode multicopper oxidases (*FET* genes), a transcription factor required for copper resistance (*SEF2*), and a phospholipid flippase (*DRS2*). There are also downregulated genes related to copper homeostasis and membrane compartments such as *CFL1*, *FRE7*, *CRP1*, *CTR1*, *CAN1*, *SUR7*, *PIL1*, and *LSP1*, encoding ferric reductase and cupric reductase *(CFL1* and *FRE7*), copper transporters (*CRP1* and *CTR1*), components of the membrane compartment occupied by Can1 (MCC) domain (*CAN1* and *SUR7*), and eisosome-associated proteins (*PIL1*, and *LSP1*) (Table S2 and Figure S1) [27,48,49,50,51,52].

To further characterize the biological functions and cellular processes associated with these DEGs, GO enrichment analysis was performed. All enriched GO terms derived from upregulated and downregulated DEGs under both copper conditions are summarized in Table S3. Notably, upregulated DEGs under high-copper conditions were enriched for metabolism and mitochondrial-related functions, including “metabolic process,” “primary metabolic process,” “macromolecule metabolic process,” “mitochondrion organization,” “mitochondrial respiratory chain complex assembly,” “mitochondrial translation,” and “mitochondrial ATP synthesis coupled electron transport” (biological process [BP] terms in Table 2 and Table S3). In contrast, downregulated DEGs under high-copper conditions were enriched for GO BP terms associated with chemical response and cellular homeostasis, including “response to chemical,” “cellular response to chemical stimulus,” “chemical homeostasis,” “intracellular chemical homeostasis,” and “transition metal ion binding” (Table 2 and Table S3). Given that copper serves as an essential cofactor for ferroxidases involved in iron uptake, this enrichment further suggests that Ndt80 may influence iron homeostasis during copper stress (Table S3). Additionally, enrichment of “oxidoreductase activity” and “oxidoreductase complex” among upregulated DEGs under both low- and high-copper conditions further links Ndt80 to oxidative stress regulation (Table S3). These findings suggest that Ndt80 plays an important role in maintaining metal homeostasis and coordinating stress responses.

Finally, Gene Set Enrichment Analysis (GSEA) was performed to examine the coordinated transcriptional changes at the pathway level. Significantly enriched gene sets (defined by a *p*-value < 0.01 and a false discovery rate (FDR) *q*-value < 0.05) are listed in Table S4. Under high-copper conditions, upregulated enriched gene sets include those associated with ribosomal function and mitochondrial processes, whereas downregulated gene sets were enriched for pathways related to metabolism, nutrient transport, and plasma membrane (Table S4).

### 3.3. NDT80 Deletion Compromises Plasma Membrane Integrity Under High-Copper Stress

**Figure 4:**
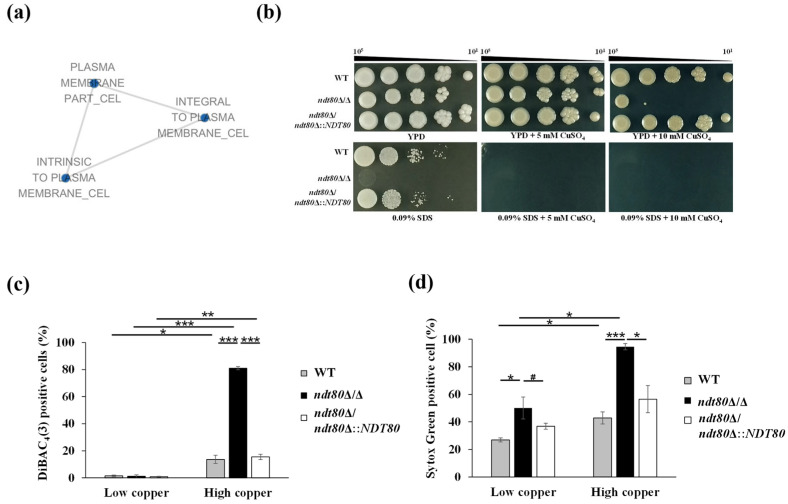
Ndt80 maintains the plasma membrane structure and permeability under copper stress. (**a**) Highly significantly enriched plasma membrane-related gene categories identified by gene set enrichment analysis (GSEA). The interaction network was generated using Cytoscape with a *p*-value < 0.01 and a FDR *q*-value < 0.05. Blue nodes represent downregulated gene sets. Node shade indicates the significant number of normalized enrichment score (NES), and node size is proportional to the total number of genes within each gene set. Gene sets with overlapping genes were grouped together to form the network. (**b**) Cell growth on YPD agar plates containing either 5 or 10 mM CuSO_4_ in the presence or absence of plasma membrane-disrupting agent SDS. (**c**) Plasma membrane potential was measured by DiBAC_4_(3) staining and analyzed by flow cytometry. (**d**) Plasma membrane permeability measured by SYTOX Green staining and analyzed by flow cytometry. Data were obtained from three independent experiments and are presented as the mean ± SD. ^#^ *p* < 0.1; * *p* < 0.05; ** *p* < 0.01; *** *p* < 0.001.

The plasma membrane serves as a critical barrier protecting cells from environmental stress. In *C. albicans*, the plasma membrane MCC/Eisosome domain has been shown to contribute to copper resistance [51,52]. The MCC domain consists of specific plasma membrane proteins and is associated with the eisosome complex [46]. Notably, our RNA-seq data revealed that under high-copper conditions, the *ndt80*Δ/Δ mutant exhibited altered expression of multiple genes encoding MCC and eisosome components, including *CAN1*, *SUR7*, *PIL1*, and *LSP1* (Table S2). In addition, the phospholipid flippase gene *DRS2*, which is required for maintaining plasma membrane phospholipid asymmetry, was also differentially expressed. Consistent with these findings, GSEA identified the downregulation of plasma membrane-associated gene sets under high-copper conditions (Table S4), which were further visualized in the interaction network (Figure 4a). These transcriptomic data suggest that Ndt80 may influence plasma membrane organization during copper stress.

To test this possibility, plasma membrane integrity was assessed using the plasma membrane-disrupting agent sodium dodecyl sulfate (SDS). SDS is an amphiphilic detergent that solubilizes lipid bilayers and disrupts the plasma membrane structure [53,54]. The *ndt80*Δ/Δ mutant displayed increased susceptibility to SDS, indicating impaired plasma membrane integrity in the absence of Ndt80. Importantly, copper supplementation further exaggerated this vulnerability, linking the role of Ndt80 in plasma membrane maintenance to cellular adaptation under elevated copper conditions (Figure 4b).

Plasma membrane potential and permeability were further assessed using DiBAC_4_(3) and SYTOX Green staining under high-copper conditions. DiBAC_4_(3), a membrane-penetrating dye, increases fluorescence upon entering depolarized cells [55]. Flow cytometric analysis revealed significantly elevated DiBAC_4_(3) fluorescence in the *ndt80*Δ/Δ mutant, indicating enhanced membrane depolarization under copper stress (Figure 4d). Similarly, SYTOX Green, which penetrates cells with compromised membranes and fluoresces upon nucleic acid binding [56], showed increased fluorescence in the *ndt80*Δ/Δ mutant, reflecting elevated membrane permeability (Figure 4d). Collectively, these results demonstrate that deletion of *NDT80* disrupts plasma membrane integrity and organization under high-copper stress, providing a mechanistic explanation for the heightened copper sensitivity observed in the *ndt80*Δ/Δ mutant.

### 3.4. Deletion of NDT80 Leads to Accumulation of Reactive Oxygen Species (ROS) and Induces Oxidative Stress Responses Under High-Copper Conditions

**Figure 5:**
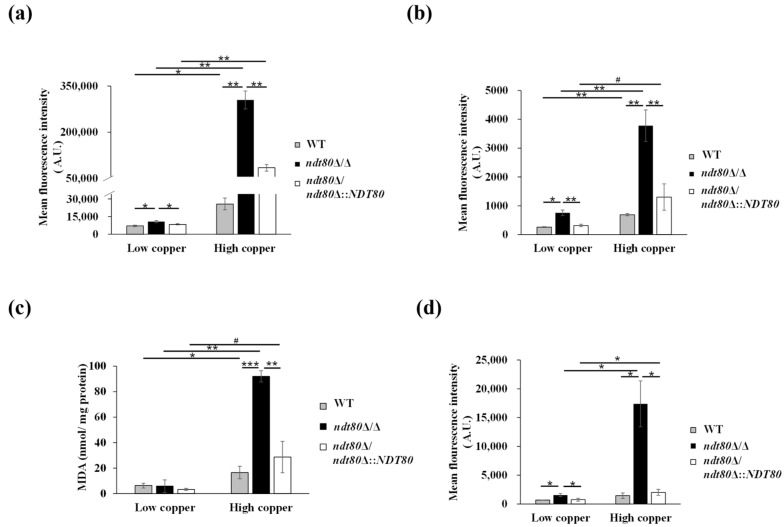
Ndt80 regulates intracellular ROS accumulation and lipid peroxidation under copper stress. Intracellular ROS levels were measured by (**a**) DHE and (**b**) H_2_DCFDA staining and analyzed by flow cytometry. (**c**) Lipid peroxidation was evaluated by measuring intracellular malondialdehyde (MDA) levels using a TBARS assay after 5 h of subculture, with or without 10 mM CuSO_4_. (**d**) Lipid droplet accumulation was assessed by Nile red staining and analyzed by flow cytometry. Data represent the mean ± SD from three independent experiments. ^#^ *p* < 0.1; * *p* < 0.05; ** *p* < 0.01; *** *p* < 0.001.

Copper plays essential roles in numerous biological processes but can also participate in redox reactions that promote the generation of ROS. Because copper homeostasis is disrupted in the *ndt80*Δ/Δ mutant under high-copper conditions (Figure 1a,b), this raises the possibility that intracellular ROS levels may be altered in this mutant strain. ROS production was thus assessed using DHE and H_2_DCFDA staining. DHE detects superoxide anions and emits red fluorescence upon oxidation and DNA binding [57,58]. H_2_DCFDA is a widely used nonfluorescent ROS indicator that, after entering cells, is deacetylated by intracellular esterases to form 2′-7′-dichlorodihydrofluorescein (DCFH_2_), which is subsequently oxidized by ROS to the highly fluorescent 2′,7′-dichlorofluorescein (DCF) [57]. As shown in Figure 5a and 5b, the *ndt80*Δ/Δ mutant exhibited significantly elevated superoxide and total ROS levels under high-copper conditions compared with the WT and *NDT80*-reintegrated strains.

Excessive ROS accumulation can trigger lipid peroxidation, leading to damage of cellular and organelle membranes [59,60]. In *C. albicans*, under oxidative stress, flavodoxin-like proteins (FLPs), including Pst1, Pst2, Pst3, and Ycp4, localize to eiosomes and protect plasma membrane lipids by inhibiting the formation of toxic semiquinone radicals [49,61,62,63]. Because *NDT80* deletion affected FLP gene expression in the RNA-seq analysis (Table S2) and compromised plasma membrane integrity (Figure 4b–d), we further investigated the relationship between ROS accumulation and lipid peroxidation under high-copper conditions. Lipid peroxidation was evaluated by measuring malondialdehyde (MDA), a well-established marker of oxidative damage. The *ndt80*Δ/Δ mutant displayed significantly higher MDA levels under high-copper conditions than the control strains (Figure 5c).

In response to oxidative and copper stresses, lipid droplets derived from the endoplasmic reticulum (ER) accumulate to sequester excess lipids and protect cellular membranes from peroxidative damage [64,65,66]. Lipid droplets also function as hubs linking membrane-bound organelles with intracellular lipid buffering [64,65,66]. To further assess oxidative stress in the *ndt80*Δ/Δ mutant, lipid droplet accumulation was examined using Nile red staining [67]. As shown in Figure 5d, markedly increased lipid droplet accumulation was observed in the *ndt80*Δ/Δ mutant under copper stress, compared with the control strains. Together, these results indicate enhanced ROS production, elevated lipid peroxidation, and increased lipid droplet accumulation in the absence of *NDT80*, which is consistent with heightened oxidative stress and plasma membrane damage under high-copper conditions.

**Figure 6:**
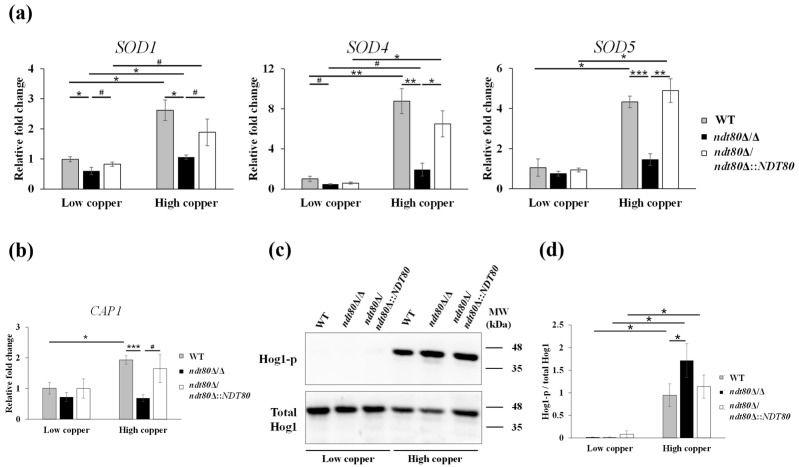
Participation of Ndt80 in copper-induced oxidative stress responses. (**a**) Expression of copper-dependent *SOD* genes following treatment with 10 mM CuSO_4_. (**b**) Expression of the oxidative stress-responsive transcription factor *CAP1* following treatment with 10 mM CuSO_4_. (**c**) Hog1 phosphorylation levels were detected by Western blotting and quantified using ImageJ software. Total Hog1 was used as the loading control to calculate the phosphorylation ratio. (**d**) Ratio of phosphorylated Hog1 (Hog1-p) to total Hog1. Data are from three independent experiments and presented as the mean ± SD. ^#^ *p* < 0.1; * *p* < 0.05; ** *p* < 0.01; *** *p* < 0.001.

Enzymatic antioxidants play central roles in mitigating oxidative stress. In *C*. *albicans*, superoxide dismutases (Sods) utilize copper-zinc (Sod1), copper-only (Sod4, Sod5, and Sod6), or manganese (Sod2 and Sod3) as cofactors to detoxify superoxide anions [3,68]. Copper availability is also crucial for Sod activation through metal cofactor exchange [69]. Given the intracellular copper accumulation and increased ROS levels observed in the *ndt80*Δ/Δ mutant under high-copper conditions (Figure 1b and Figure 5a,b), we next examined whether the expression of *SOD* genes was affected. Real-time qPCR analysis revealed that expression of the copper-dependent *SOD* genes *SOD1*, *SOD4*, and *SOD5* was significantly reduced in the *ndt80*Δ/Δ cells under the high-copper conditions (Figure 6a), whereas expression of *SOD2*, *SOD3,* and *SOD6* remained unchanged (Figure S2). These findings suggest that Ndt80 positively regulates copper-dependent antioxidant defenses, thereby contributing to protection against copper-induced oxidative stress.

The transcription factor Cap1 is a major regulator of antioxidant gene expression, including *SODs* [70,71,72]. In addition, the Hog1 mitogen-activated protein kinase (MAPK) pathway functions independently of Cap1 to mediate oxidative stress responses in *C. albicans* [70,73]. To further dissect the signaling pathways involved, *CAP1* expression was examined under copper stress. *CAP1* transcript levels were induced in the WT and *NDT80*-reintegrated strains under high-copper conditions compared with low-copper conditions (Figure 6b). In contrast, *CAP1* expression in the *ndt80*Δ/Δ mutant was lower and showed no significant induction in response to elevated copper (Figure 6b). Notably, phosphorylation of Hog1 was substantially increased in the *ndt80*Δ/Δ mutant relative to the control strains under high-copper conditions (Figure 6c,d). These results indicate that, in the absence of Ndt80, impaired Cap1-mediated antioxidant responses coincide with the enhanced activation of Hog1 signaling, suggesting that these two regulators participate in Ndt80-associated oxidative stress regulation under copper stress.

### 3.5. Ndt80 Deletion Impairs Mitochondrial Respiration Under High-Copper Conditions

**Figure 7:**
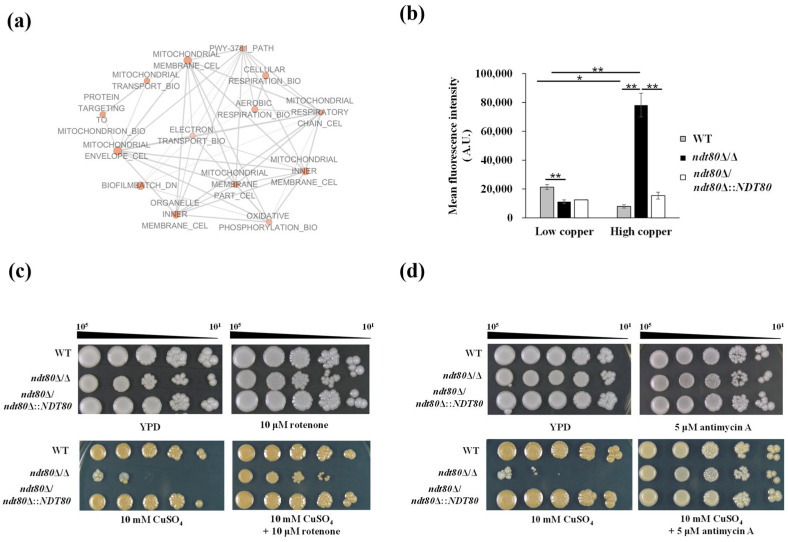
Loss of *NDT80* promotes mitochondrial ROS production through enhanced mitochondrial ETC activity. (**a**) Significantly enriched mitochondrial gene categories identified by gene set enrichment analysis (GSEA). The interaction network was generated using Cytoscape with a cutoff of *p* < 0.01 and an FDR *q*-value < 0.05. Red nodes represent upregulated gene sets. Node shade indicates the significant number of normalized enrichment score (NES), and node size is proportional to the total number of genes within each gene set. Gene sets with overlapping genes were grouped together to form the network. (**b**) Mitochondrial ROS levels under high-copper conditions owere assessed by MitoSox Red staining and analyzed by flow cytometry. Mean fluorescence intensity (MFI) is shown. Data represent the mean ± SD from three independent experiments. * *p* < 0.05; ** *p* < 0.01. (**c**) Growth of cells on YPD plates containing 10 mM CuSO_4_ in the presence or absence of the mitochondrial ETC complex I inhibitor rotenone (10 μM). (**d**) Growth of cells on YPD plates containing 10 mM CuSO_4_ in the presence or absence of the mitochondrial ETC complex III inhibitor antimycin A (5 μM). Results in (**c**,**d**) are representative of three independent experiments. Plates were photographed after 3 d of incubation at 30 °C.

Excess copper induces dysfunction in multiple organelles by promoting oxidative stress and protein damage [11,74,75,76]. Mitochondria are particularly sensitive to fluctuations in copper levels. Cytochrome *c* oxidase, the terminal enzyme of the ETC, requires copper as an essential cofactor to facilitate electron transfer. Proper cytochrome *c* oxidase activity is, therefore, critical for maintaining mitochondrial redox balance and ATP production during respiration [77,78]. However, copper overload disrupts mitochondrial function by impairing respiratory activity, increasing membrane permeability, and dissipating the mitochondrial membrane potential (ΔΨ). In addition, excess copper overload stimulates mitochondrial ROS production, leading to oxidative damage to mitochondrial proteins and membranes [79]. Because RNA-seq analysis revealed significant enrichment of mitochondrial- and respiration-related gene sets in the *ndt80*Δ/Δ mutant under high-copper conditions (enriched GO terms in Table 2 and GSEA results in Figure 7a), we further investigated the role of Ndt80 in regulating mitochondrial respiration and ETC activity during copper stress.

Mitochondrial respiration represents a major source of intracellular ROS, primarily generated at ETC complexes I and III. Copper overload exacerbates oxidative damage, thereby further compromising mitochondrial structure and function. To determine whether excessive mitochondrial ROS contributes to copper sensitivity in the *ndt80*Δ/Δ mutant (Figure 1a), mitochondrial ROS levels were measured using MitoSox Red staining (Figure 7b). MitoSox Red, a positively charged derivative of DHE, selectively accumulates in mitochondria and emits red fluoresces upon oxidation by superoxide [57]. Flow cytometric analysis revealed significantly elevated mitochondrial ROS levels in the *ndt80*Δ/Δ mutant under high-copper conditions compared with the WT and *NDT80*-reintegrated strains, indicating that Ndt80 plays an important role in maintaining mitochondrial ROS homeostasis during copper stress (Figure 7b).

To further assess whether ETC-derived ROS contributes to copper toxicity, the activities of complexes I and III were inhibited using rotenone and antimycin A, respectively (Figure 7c,d). As expected, the *ndt80*Δ/Δ mutant exhibited pronounced growth defects under high-copper conditions, consistent with Figure 1a. Notably, inhibition of ETC complexes markedly alleviated copper-induced growth inhibition in the *ndt80*Δ/Δ mutant. These findings support the conclusion that excess mitochondrial ROS generated from the ETC contributes to copper sensitivity in the absence of Ndt80.

**Figure 8:**
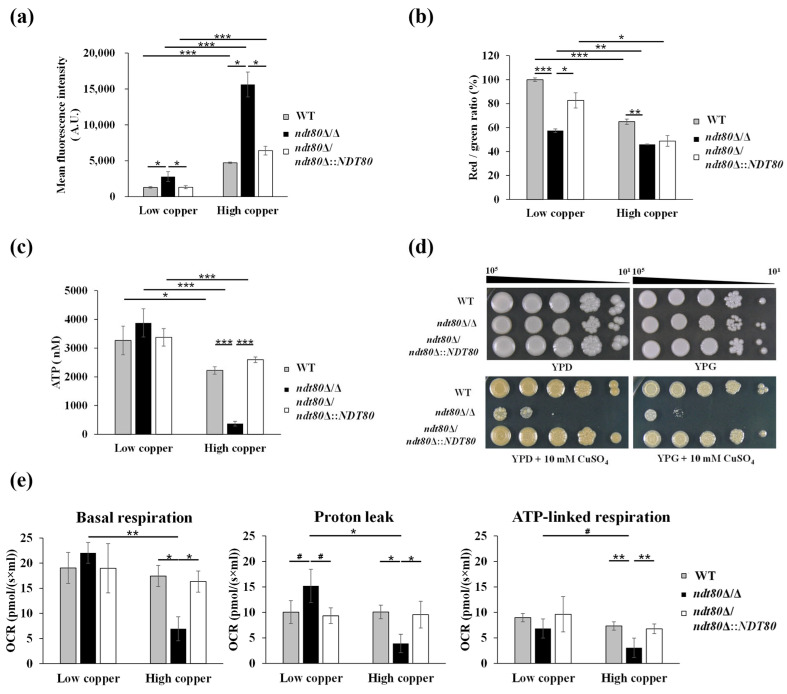
Mitochondrial ROS generated by the ETC under high-copper conditions disrupts mitochondrial membrane potential (ΔΨ) and ATP production. (**a**) Mitochondrial ΔΨ was assessed using Rhodamine 123 staining. Mean fluorescence intensity (MFI) was analyzed by flow cytometry. (**b**) Mitochondrial ΔΨ was evaluated by JC-1 staining and expressed as the red-to-green fluorescence ratio. Mean fluorescence intensities of red and green signals were analyzed by flow cytometry. (**c**) Intracellular ATP levels were determined using a luminescence-based assay. (**d**) Cell growth on plates containing 10 mM CuSO_4_ with different carbon sources: YPD (glucose) and YPG (glycerol). (**e**) Oxygen consumption rate (OCR) was measured by high-resolution respirometry using the Oroboros O2k system following copper treatment. Data represent the mean ± SD from three independent experiments. ^#^ *p* < 0.1; * *p* < 0.05; ** *p* < 0.01; *** *p* < 0.001.

The elevated mitochondrial ROS observed in the *ndt80*Δ/Δ mutant (Figure 7b) suggested underlying mitochondrial dysfunction. To further evaluate mitochondrial integrity, mitochondrial membrane potential (ΔΨ), a key indicator of mitochondrial health, was examined using Rhodamine 123 and JC-1 staining (Figure 8a,b). Rhodamine 123 is a lipophilic and cationic dye that accumulates in polarized mitochondria; depolarization leads to dye release and green fluorescence [80]. Similarly, JC-1 forms red fluorescence aggregates in polarized mitochondria, whereas depolarized mitochondria display green fluorescence due to monomeric JC-1 [81]. Flow cytometric analysis demonstrated that even under low-copper conditions, the *ndt80*Δ/Δ mutant exhibited a higher proportion of depolarized mitochondria compared with the control strains. Under high-copper conditions, mitochondrial dysfunction was further exacerbated, with a significantly greater loss of ΔΨ in the *ndt80*Δ/Δ mutant (Figure 8a and 8b). These results indicate that Ndt80 is required to maintain mitochondria ΔΨ during copper stress. Because mitochondrial membrane depolarization compromises ATP synthesis [82], intracellular ATP levels were subsequently measured. Under high-copper conditions, ATP levels were significantly reduced in the *ndt80*Δ/Δ mutant relative to the WT and *NDT80*-reintegrated strains (Figure 8c), confirming that mitochondrial dysfunction in the absence of *NDT80* leads to impaired cellular energy production.

*C. albicans* is a Crabtree-negative yeast that preferentially relies on mitochondrial respiration rather than fermentation for ATP generation, even in the presence of glucose [83,84,85]. Furthermore, within host environments where glucose availability is limited, *C. albicans* utilize nonfermentable carbon sources such as glycerol via respiration to sustain ATP production [86]. To further evaluate mitochondrial energetic capacity under copper stress, growth was assessed on glucose- and glycerol-based media supplemented with copper (Figure 8d). The *ndt80*Δ/Δ mutant exhibited pronounced copper sensitivity on both carbon sources, further supporting a role for Ndt80 in mitochondrial respiration and energy homeostasis. Finally, mitochondrial respiration capacity was directly examined by measuring the oxygen consumption rate (OCR) (Figure 8e). Following copper treatment, the *ndt80*Δ/Δ mutant displayed a significantly reduced basal respiration compared with the WT and *NDT80*-reintegrated strain. Moreover, both proton leak and ATP-linked respiration were markedly diminished in the *ndt80*Δ/Δ mutant under high-copper conditions. Collectively, these findings demonstrate that Ndt80 is essential for maintaining mitochondrial respiration, redox balance, and ATP production during copper stress.

## 4. Discussion

To resist cytotoxic copper levels in host niches and evade copper-induced oxidative bursts from immune cells, *C. albicans* exhibit significantly higher copper tolerance than the model yeast *S. cerevisiae*, which encounters comparatively limited copper stress in its rotting-fruit habitat [18,19,87]. Copper tolerance in *C*. *albicans* is, therefore, achieved through multiple adaptive mechanisms and strict regulatory systems governing cellular copper homeostasis. However, current knowledge of copper tolerance in *C. albicans* remains largely inferred from the relatively simple system of *S. cerevisiae* and a limited number of studies focusing on the copper-responsive transcription factor Cup2 [7,10,13,75]. In this study, we investigated copper tolerance mechanisms in *C. albicans*, with a particular emphasis on regulation mediated by the transcription factor Ndt80.

Previous studies have demonstrated that Ndt80 plays critical roles in hyphal development, biofilm formation, nitric oxide detoxification in macrophages, antifungal drug resistance, and virulence in *C. albicans* [24,25,26,28]. Its broad regulatory capacity is supported by the extensive promoter-binding profile, which expands the transcriptional scope of Ndt80-dependent regulation [25]. Notably, our results (Figure 1a,b) show that Ndt80 responds to cytotoxic copper levels and influences intracellular copper accumulation, revealing a novel link between Ndt80 and copper stress response. Furthermore, gene expression levels from qPCR, GO analysis of RNA-seq data, together with the identification of putative Ndt80-binding motifs within promoter regions of many copper-responsive DEGs, support the involvement of Ndt80 in copper homeostasis and stress adaptation (Figure 2, Tables S3 and S5). Finally, in addition to Ndt80, at least two other transcription factors can regulate copper-responsive genes. Mac1 and Cup2 play roles in the expression of these genes under copper-deprived and high-copper conditions, respectively [48,50]. The relationship between Ndt80, Mac1, and Cup2 will be interesting for future study.

The plasma membrane represents a crucial physical barrier that protects cells from environmental stresses, including copper toxicity [51]. In addition, internalization of the plasma membrane copper transporter Ctr1 via endocytosis is critical for preventing copper overload under excess copper conditions [88]. RNA-seq and GSEA revealed the downregulation of genes associated with plasma membrane and membrane phospholipids in the *ndt80*Δ/Δ mutant, suggesting that Ndt80 contributes to plasma membrane lipid homeostasis during copper stress (Table S2 and Figure 4a). Consistently, copper-induced impairment of plasma membrane potential and increased membrane permeability were observed in the absence of Ndt80 (Figure 4c,d), further linking Ndt80 to the maintenance of plasma membrane integrity under copper stress (Figure 4c,d). Notably, previous studies have shown that Ndt80 modulates ergosterol biosynthesis through the regulation of *ERG* genes, thereby protecting the plasma membrane from fluconazole-induced damage. These findings raise the possibility that ergosterol biosynthesis may also represent a mechanism through which Ndt80 reserves membrane stability during copper exposure [25,89].

In addition to extracellular defense mechanisms, *C. albicans* employ intracellular strategies to mitigate copper toxicity. Copper metallothioneins constitute a primary system that sequesters excess cytosolic copper and prevents oxidative damage and protein destabilization caused by copper-induced ROS [10,12,13]. Because intracellular metal availability influences cofactor switching of superoxide dismutases [69], our results indicate that Ndt80 may activate expression of copper-dependent *SOD*s under high-copper conditions (Figure 6a), thereby limiting intracellular ROS accumulation in wild-type cells (Figure 5a,b). These findings reveal a previously uncharacterized role of Ndt80 in coordinating oxidative stress responses during copper stress.

Copper exerts a dual effect on mitochondrial function: it is essential for electron transfer by cytochrome *c* oxidase, yet excessive copper disrupts respiration through protein damage and mitochondrial membrane depolarization [4,78,90,91]. Intriguingly, RNA-seq analysis revealed the upregulation of mitochondrial respiration under high-copper conditions (Table 2 and Figure 7a), suggesting that Ndt80 influences mitochondrial responses to copper stress. Consistently, mitochondrial ROS assays demonstrated that the loss of Ndt80 resulted in excessive ROS accumulation within mitochondria following copper exposure (Figure 7b). Moreover, inhibition of ETC complexes I and III, major sites of ROS generation, significantly alleviated copper sensitivity in the *ndt80*Δ/Δ mutant (Figure 7c,d). These results indicate that overactivation of mitochondrial respiration in the absence of Ndt80 may drive ROS overproduction, ultimately leading to mitochondrial dysfunction under high-copper conditions.

Although enhanced respiration is generally associated with increased ATP synthesis, intracellular ATP levels were markedly reduced in the *ndt80*Δ/Δ mutant (Figure 8c). A possible explanation is the collapse of the mitochondrial membrane potential (ΔΨ), as demonstrated in Figure 8a,b. Efficient ATP production requires both intact electron transfer and maintenance of the proton gradient across the mitochondrial inner membrane. Without sufficient ΔΨ, upregulation of ETC components alone, as listed in Table 2, is insufficient to sustain ATP synthesis. Reduced ATP availability may further impair the activity of the P-type ATPase copper exporter Crp1, potentially explaining the activation of *CRP1* expression despite defective copper extrusion in the *ndt80*Δ/Δ mutant. (Figure 1b and Figure 2). Thus, copper overload in the absence of Ndt80 disrupts mitochondrial function at both the transcriptional and bioenergetic levels.

The combined effects of Ndt80 on plasma membrane integrity and mitochondrial homeostasis under high-copper conditions suggest a broader regulatory role of Ndt80 in the endomembrane system. Supporting this notion, RNA-seq analysis revealed altered expression of genes encoding MCC and the eisosome components in the *ndt80*Δ/Δ mutant (Table S2), further implicating Ndt80 in endocytosis and membrane organization during copper stress. Notably, copper has been reported to synergistically enhance the antifungal activity of fluconazole, a phenomenon that may be related to Ndt80-mediated regulation of ergosterol biosynthesis and copper homeostasis [25,92,93]. Although further investigation is required, this study provides new insights into copper tolerance mechanisms and uncovers previously unappreciated functions of Ndt80 in regulating membrane integrity, mitochondrial respiration, and metal homeostasis in *C. albicans*.

## Figures and Tables

**Table 1:** *C. albicans* strains used in this study.

Strain	Genotype	Comment	Reference
SC5314	*NDT80*/*NDT80*	WT	[28]
YLO386	*ndt80*Δ-*FRT*/*ndt80*Δ-*FRT*	*NDT80* deletion (*ndt80*Δ/Δ)	[28]
YLO464	*ndt80*Δ-*FRT*/*ndt80*Δ-*FRT*::*NDT80*-*FRT*	*NDT80* reintegration (*ndt80*Δ/*ndt80*Δ::*NDT80*)	[28]

**Table 2:** Representative mitochondria- and metal-stress-related GO terms enriched among DEGs under high-copper conditions.

GO ID	GO Terms from the Upregulated DEGs ^a^
0007005	Mitochondrion organization (BP)
0140053	Mitochondrial gene expression (BP)
0072655	Establishment of protein localization to mitochondrion (BP)
0033108	Mitochondrial respiratory chain complex assembly (BP)
0070585	Protein localization to mitochondrion (BP)
0032543	Mitochondrial translation (BP)
0042775	Mitochondrial ATP synthesis coupled electron transport (BP)
0042773	ATP synthesis coupled electron transport (BP)
0030150	Protein import into mitochondrial matrix (BP)
0005739	Mitochondrion (CC)
0005740	Mitochondrial envelope (CC)
0031966	Mitochondrial membrane (CC)
0005743	Mitochondrial inner membrane (CC)
0005759	Mitochondrial matrix (CC)
0098798	Mitochondrial protein-containing complex (CC)
0005761	Mitochondrial ribosome (CC)
0098803	Respiratory chain complex (CC)
0005762	Mitochondrial large ribosomal subunit (CC)
1990204	Oxidoreductase complex (CC)
0005763	Mitochondrial small ribosomal subunit (CC)
**GO ID**	**GO Terms from the Downregulated DEGs**
0042221	Response to chemical (BP)
0070887	Cellular response to chemical stimulus (BP)
0048878	Chemical homeostasis (BP)
0055082	Intracellular chemical homeostasis (BP)
0006879	Intracellular iron ion homeostasis (BP)
0046914	Transition metal ion binding (MP)

^a^ Three major categories in Gene Ontology (GO): BP: biological process; MP: molecular process; CC: cellular component.

## Data Availability

The original contributions presented in this study are included in the article/Supplementary Material. Further inquiries can be directed to the corresponding authors.

## Supplementary Materials

The following supporting information can be downloaded at: https://www.mdpi.com/article/10.3390/jof12040294/s1. Table S1. Primers used in this study. Table S2. List of differentially expressed genes (DEGs). Table S3. Gene ontology (GO) terms from the DEGs. Table S4. Enriched gene sets revealed by Gene Set Enrichment Analysis (GSEA). Table S5. Ndt80-binding motifs present in promoters of copper-related DEGs. Figure S1. Expression of SOD genes was uninterrupted by Ndt80 following treatment with 10 mM CuSO4. Gene expression levels were determined by real-time qPCR. Results were collected from three independent experiments and are presented as the mean ± SD. * *p* < 0.05. References [28,38,39,40] are cited in Supplementary Materials.
