# Supplementary material for: Ndt80 Orchestrates Copper Stress Responses and Mitochondrial Homeostasis in Candida albicans
Source: J Fungi (Basel). 2026 Apr 20;12(4):294. doi: 10.3390/jof12040294 (PMC13117858; doi:10.3390/jof12040294)
Supplement: Supplementary file 1 [file jof-12-00294-s001.zip › Supplementary File Chen et al.pdf]

Supplementary Tables

Table S1. Primers used in this study.

Primer	Sequences (5' to 3')
CTR1-F	CCGAAGTAGAACAAGGGATTTCTC
CTR1-R	GGATTTGACGGGTGGTGATT
CRP1-F	CCAGTGCTGCTATGGCTTTG
CRP1-R	GGTGAAAGCTCACTCTCGGT
SOD1-F	TCCGAATCCGCTCCAACCACA
SOD1-R	AAATGAGGACCAGCAGAAGTACAACCA
SOD2-F	TCAATTGAACAAGCCGTTGAAGCCAAA
SOD2-R	ACCACCTTGAGAGACAGGAGCCA
SOD3-F	CAATGCCGCTATTGACGCACTTGA
SOD3-R	TCCAGAACAAACTGTGGTTGGTGTGT
SOD4-F	TTTGAGCCAGCAAACAATGG
SOD4-R	CACCTGAAGGCAATCCAGTTAAA
SOD5-F	AAGGATTGCCCTCTGATATTGG
SOD5-R	GATGCTGGCACTGGTTTTTCA
SOD6-F	GGTGGTGTTCCATTATCCGA
SOD6-R	ATCGGTTTGGGTGTATTCGT
CAP1-F	TGACAACTTTGATCTTGCATTGG
CAP1-R	TGGATCGGCTTCTGCTTCAT

Table S2. List of differentially expressed genes (DEGs)

Table S2 includes DEGs from RNA-seq analysis, comparing the *ndt80*Δ/Δ and wild-type strains in YPD medium ("low copper") and YPD supplemented with 10 mM $CuSO_4$ ("high copper"), respectively. Table S2 is provided in a separate Excel file.

Table S3. Gene ontology (GO) terms from the DEGs.

Table S3 includes the GO analysis results from all the DEGs listed in Table S2, and is provided in a separate Excel file.

Table S4. Enriched gene sets revealed by Gene Set Enrichment Analysis (GSEA).

Table S4 includes the GSEA results from RNA-seq analysis in comparison of the *ndt80*Δ/Δ and wild-type strains in YPD medium alone ("low copper") and YPD supplemented with 10 mM $CuSO_4$ ("high copper"), respectively. Table S4 is provided in a separate Excel file.

Table S5. Ndt80-binding motifs present in promoters of copper-related DEGs

Gene name	Ndt80-binding motifs[a]
FET31	aaaaGGACACAAAAAAaatc(-981R)
	aggaCACAAAaaaa(--984R)
	gttaCACAAAtaga(-710R)
FET33	gcaaCACAAAcaac(-813F)
	aaggCACAAAaaaa(-170F)
SEF2	ctgcCACAAAagaa(-469F)

	tgcaCGCAAAatat(-354F)
	cacaCACAAAtata(-70F)
	caccCACAAAaccg(-372R)
DRS2	tcacCACAAAtcat(-547R)
	aatcCACAAAaagc(-768F)
CFL1	ttttCACAAAatga(-281F)
	ccctCGCAAAca(-8F)
FRE7	ttacCACAAAacgt(-776R)
	tgagCACAAAacgt(-653R)
	TaatGGACACAAAAATagat(-502F)
CRP1	tggaCACAAAaata(-499F)
	attaCACAAAagca(-146F)
	tatcCGCAAAATCT(-667R)
	taatCACAAAacac(-984F)
CTR1	aaaaCACAAAattt(-977F)
	aaacCACAAAcaca(-354F)
CAN1	atctCACAAAagct(-938F)
SUR7	tataTTTCACAAAAAAGaaga(-470F)
	tgttCACAAAattt(-152F)
	tataTTACACAAAACCaact(-579R)
PIL1	caatCACAAAgtac(-190F)
	attaCACAAAacca(-582R)
LSP1	caatCACAAAgaaa(-474R)
	cttaTTACACAAAAACagaa(-280F)
CRD2	attaCACAAAaaca(-277F)
	aactCACAAAgaga(-195F)
	ttcccCACAAAtttt(-124F)
	attaGACGCAAAAcaag(-739D)
	tagaCGCAAAacaa(-737F)
SOD4	aactCACAAAtgaa(-590F)
	gttaCGCAAAacga(-349F)
	ctccCACAAAtcaa(-304F)
	ttagCACAAAacat(-144R)
SOD5	tgacCACAAAcata(-455F)

[a]Ndt80-binding motifs are derived from the following studies:

1. Yang, Y.L.; Wang, C.W.; Leaw, S.N.; Chang, T.P.; Wang, I.C.; Chen, C.G.; Fan, J.C.; Tseng, K.Y.; Huang, S.H.; Chen, C.Y.; et al. R432 is a key residue for the multiple functions of Ndt80p in *Candida albicans*. *Cell. Mol. Life Sci.* **2012**, 69, 1011–1023. https://doi.org/10.1007/s00018-011-0849-5. (also listed as reference #28 in the main text)

2. Chen C.G.; Yang Y.L.; Shih H.I.; Su C.L.; Lo H.J. CaNdt80 is involved in drug resistance in *Candida albicans* by regulating *CDR1*. *Antimicrob. Agents Chemother*. **2004**, 48, 4505-4512. https://doi.org/10.1128/aac.48.12.4505-4512.2004. (also listed as reference #38 in the main text)

3. Ahn C.H.; Lee S.; Cho E.; Kim H.; Chung B.; Park W.; Shin J.; Oh K.B. A farnesoic acid-responsive transcription factor, Hot1, regulates yeast-hypha morphogenesis in *Candida albicans*. *FEBS Lett*. **2017,** 591,1225-1235. https://doi.org/10.1002/1873-3468.12636. (also listed as reference #39 in the main text)

4. Nobile, C.J.; Fox, E.P.; Nett, J E.; Sorrells, T.R.; Mitrovich, Q.M.; Hernday, A.D.; Tuch, B.B.; Andes, D.R.; Johnson, A.D. A recently evolved transcriptional network controls biofilm development in *Candida albicans*. *Cell.* **2012**, 148, 126–138. https://doi.org/10.1016/j.cell.2011.10.048. (also listed as reference #40 in the main text)

Supplementary figures

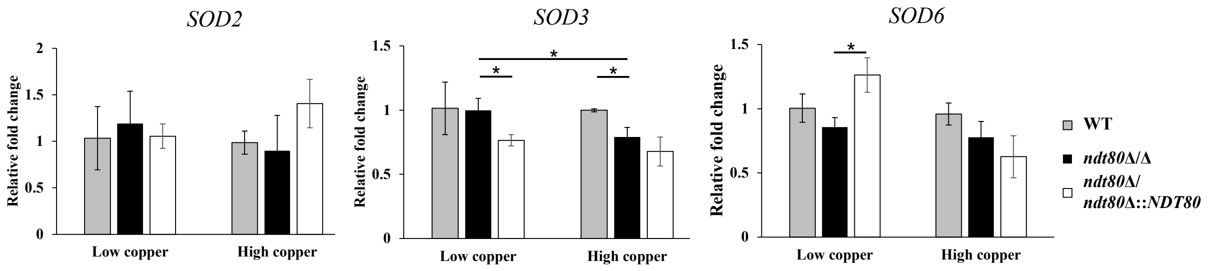

Figure S1. Expression of *SOD* genes was uninterrupted by Ndt80 following treatment with 10 mM CuSO4. Gene expression levels were determined by real-time qPCR. Results were collected from three independent experiments and are presented as the mean ± SD. *$P < 0.05$.
